# Arsenic Speciation of Contaminated Soils / Solid Wastes and Relative Oral Bioavailability in Swine and Mice

**DOI:** 10.3390/soilsystems2020027

**Published:** 2018

**Authors:** Brooke N. Stevens, Aaron R. Betts, Bradley W. Miller, Kirk G. Scheckel, Richard H. Anderson, Karen D. Bradham, Stan W. Casteel, David J. Thomas, Nicholas T. Basta

**Affiliations:** 1 School of Environment and Natural Resources, The Ohio State University, Columbus, OH 43210, United States; 2 US Environmental Protection Agency, Office of Research and Development, National Risk Management Research Laboratory, Cincinnati, OH 45224, United States; 3 US Air Force Center for Engineering and the Environment, Lackland AFB, TX 78236, United States; 4 US Environmental Protection Agency, Office of Research and Development, National Exposure Research Laboratory, Research Triangle Park, NC 27711, United States; 5 Department of Veterinary Pathobiology, University of Missouri, Columbia, MO 65211, United States

**Keywords:** arsenic, bioavailability, speciation, EXAFS, XANES

## Abstract

Arsenic (As) is one of the most widespread, toxic elements in the environment and human activities have resulted in a large number of contaminated areas. However abundant, the potential of As toxicity from exposure to contaminated soils is limited to the fraction that will dissolve in the gastrointestinal system and be absorbed into systemic circulation or bioavailable species. In part, the release of As from contaminated soil to gastrointestinal fluid depends on the form of solid phase As also termed “As speciation.” In this study, 27 As-contaminated soils and solid wastes were analyzed using X-ray absorption spectroscopy (XAS) and results were compared to *in vivo* bioavailability values determined using the adult mouse and juvenile swine bioassays. Arsenic bioavailability was lowest for soils that contained large amounts of arsenopyrite and highest for materials that contained large amounts of ferric arsenates. Soil and solid waste type and properties rather than the contamination source had the greatest influence on As speciation. Principal component analysis determined that As(V) adsorbed and ferric arsenates were the dominant species that control As speciation in the selected materials. Multiple linear regression (MLR) was used to determine the ability of As speciation to predict bioavailability. Arsenic speciation was predictive of 27% and 16% of RBA As determined using the juvenile swine and adult mouse models, respectively. Arsenic speciation can provide a conservative estimate of RBA As using MLR for the juvenile swine and adult mouse bioassays at 55% and 53%, respectively.

## 1. Introduction

Arsenic (As) is widespread across the environment. Not only is it naturally occurring in soils and geological materials, it has been used in a variety of ways by humans since ancient times. Arsenic has been used as a medicine, pesticide, herbicide, colorant, additive to animal feed, wood treatment, and as a poison [1]. Human use and the high toxicity potential has led to arsenic becoming the number one hazard of concern on the Agency for Toxic Substances and Disease Registry (ATSDR) National Priorities List (NPL) [2]. However abundant, the exposure and potential toxicity of As from contaminated soils is limited to the fraction of As that will dissolve in the gastrointestinal system and be available for absorption into systemic circulation (i.e. bioaccessible) [3]. Once bioaccessible, As can be absorbed across the intestinal epithelium and enter systemic circulation where the As is bioavailable. In part, the release of As from contaminated soil to gastrointestinal fluid depends on the form of solid phase arsenic also termed “As speciation.”

Relating As speciation to bioavailability can prove beneficial when considering site cleanup for contaminated areas. If the speciation of As within the soil limits its dissolution into the gastrointestinal system, then the potential bioavailability will be low and can result in higher cleanup levels of As in soil or not having to perform soil cleanup at all. There have been many studies that investigate the bioavailability of As contaminated soils [4]. A large number of soils have been evaluated for As bioavailability using the juvenile swine bioassay. Juvenile swine were chosen due to the gastrointestinal system similarities between swine and humans. The growth rate and subsequent parameters of juvenile swine are similar to that of children who are often identified as the most susceptible population regarding exposure to As contaminated soils [5,6]. In addition, to the juvenile swine bioassay, the U.S. Environmental Protection Agency (U.S. EPA) developed a bioassay with adult mice, which has been used to determine As bioavailability for a number of soils [7,8].

There are several excellent spectroscopic methods capable of determining arsenic speciation, but the most authoritative and direct measurement is through X-ray absorption spectroscopy (XAS). X-ray absorption spectroscopy is used to determine oxidation state, coordination environment, interatomic bond distances, and the identity of nearest-neighbor elements relative to the As soil contaminant. X-ray absorption spectroscopy experiments provide an *in situ* look at the current chemical form of soil metals that can be used to predict the long-term fate of the metal and its potential bioavailability based on known solubility products.

Several studies have attempted to relate As XAS to As solubility, but only a few to bioaccessible As and even fewer with *in vivo* animal exposure experiments (bioavailability). Brattin et al. [9] used *in vitro* bioaccessibility (IVBA) methods to predict bioavailability of soils for the juvenile swine bioassay. After a good prediction was achieved using the *in vitro* bioaccessibility method, the authors included As speciation data obtained via electron microprobe analysis for the 20 soils into the predictive equation, which increased the R^2^ value from 0.723 to 0.906 [9]. Attempts to correlate relative abundance of As species and RBA have found limited success in mouse [7] and in juvenile swine [10] animal models. In a study using the mouse bioassay, Bradham et al. [7], determined that the amount of arsenopyrite (FeAsS) was a significant predictor of bioavailable As (negatively correlated) in 11 soils. Although significant (P<0.10), the R^2^ value of 0.28 for the predictive equation indicates that the overall fit was not very good, resulting in the conclusion that As speciation does not accurately predict bioavailable As [7]. In a dataset of 19 samples, Foster et al. [11] found significant correlations between the amount of As released during IVBA and As(V) sorbed to gibbsite, As(V) substituted in jarosite (positive correlations) and arsenopyrite (negative correlation).

Among the studies correlating As XAS data with bioavailable and/or bioaccessible As, most have used mine-impacted soils [10–12]. In our study, we attempted to determine if As speciation can be predictive of As bioavailability in either the juvenile swine or adult mouse bioassays from a large (27 soils) dataset which includes arsenic from diverse contamination sources and geographic regions.

## 2. Materials and Methods

### 2.1 Soil and solid waste preparation

Twenty-seven arsenic (As) containing soils and solid wastes that represent a wide variety of As sources were collected, homogenized and sieved to <250 µm. Homogenization of the 27 soils and solid wastes was done by mixing air dried soil in an HDPE rotary mixer and analysis via microwave assisted digestion [13]. Homogenization analysis was done by dividing the materials into eight equal units then three subsamples from each unit were analyzed, resulting in a total of 24 samples. The means of each unit were calculated and analysis of variance (ANOVA) tests performed to ensure homogenization. Analysis of variance testing resulting in no difference within units and between units at a 95% confidence level (P <0.05). The units were combined into one container for laboratory use for all further analysis. One of the 27 materials was a National Institute of Standards (NIST) Standard Reference Material (SRM) 2710A (Montana Soil II) and was not homogenized because homogenization was done prior to certification at NIST. All materials were stored at 25 °C as dry powders for further analysis.

### 2.1 In Vivo Bioavailability

The *in vivo* adult mouse and juvenile swine bioassays were used to determine bioavailable As for the 27 study soils and solid wastes. The adult mouse bioassay was performed according to Bradham et al. [7,8]. The adult mouse bioassay was conducted with C57BL/6 mice and the urinary excreted fraction (UEF) of the dosed As was used to determine bioavailable As. Test soils were mixed with AIN-93G purified rodent diet obtained from Dyets (Bethlehem, PA) to a 1% (wt/wt) soil:diet ratio. Animals were allowed to consume drinking water and the mixture of test material and basal diet with unlimited access. Excreted urine was collected and stored at −20 °C until As analysis was completed. The juvenile swine bioassay was performed according to Brattin and Casteel [14]. The juvenile swine bioassay was conducted with juvenile males and the UEF of the dosed As was used to determine RBA As. Test soils were placed in the center of a ball of moistened feed that did not contain detectable amounts of As to achieve an As dose ranging from 40 – 350 ug/kgBW-day. Feed balls containing test material were given to the animals twice daily. Dosing occurred 2 hours prior to feeding ensuring the animals were in a semi-fasted state and to limit any interactions due to food on As absorption. Although test material and feed was limited, animals had unlimited access to drinking water that did not contain detectable amounts of As. Excreted urine was collected, acidified with nitric acid, and refrigerated until As analysis was completed.

Relative bioavailability (RBA) for both bioassays was calculated as the ratio of the As UEF for a test material to the As UEF in a diet containing a reference arsenical (e.g., sodium arsenate, Na_3_AsO_4_) (Equation 1).

(1) $$RBA\;\%=\frac{UEF\;\%\;Soil}{UEF\;\%\;Na_{3}AsO_{4}}$$

### 2.2 Arsenic Speciation Methods

X-ray absorption spectroscopy was performed on all 27 soils and solid wastes at the Materials Research Collaborative Access Team (MRCAT) beamline 10-BM, Sector 10, at the Advanced Photon Source of the Argonne National Laboratory, U.S. The storage ring operated at 7 GeV in top-up mode. A liquid N_2_ cooled double crystal Si(111) monochromator was used to select the incident photon energies and a platinum-coated mirror was used for harmonic rejection.

Each of the soil and solid waste materials as prepared for bioassays were further prepared by fracturing with a mortar and pestle, pressing into a 1-cm pellet, and encasing in Kapton tape. Standard material dilutions for sample preparation were determined by XAFSMass (Klementiev, 2012). Data collection was conducted in transmission and fluorescence modes (Vortex-ME4, silicon drift detector, SII) with several layers of aluminum foil covering the fluorescence detector window to suppress fluorescence from other elements (such as iron) in the samples. Up to five As K*α* (11867 eV) spectra were collected in transmission and fluorescence mode at room temperature for every soil. Each scan included simultaneous collection of transmission on a reference sodium arsenate powder for energy calibration at the first derivative inflection point (11874 eV).

Background subtraction and calibration were performed in the Athena module of Demeter software [15]. Each scan was calibrated to a sodium arsenate (As(V)) standard (11874 eV), averaged, normalized, and the background was removed by spline fitting [15]. The normalized X-ray absorption near edge structure (XANES) were used in linear combination fitting (LCF) models for relative abundance of oxidation state resolved by spectral edge position (e0 energy). Methods and table of standards for XANES LCF are in Table S1 in Supplemental Information.

Species identification was performed in a two-step process using SIXpack software for principle component analysis (PCA) [16] and the IFEFFIT software package for linear combination fitting (LCF)[15] using the extended X-ray absorption fine structure (EXAFS) with a k^3^-weighting. PCA was used to determine the number of components (standards) that are most likely to be present in the samples and target transformation was used to identify the most suitable standards for LCF. Standards used and their SPOIL values are provided in Supplemental Table S2.

Linear combination model quality was defined by smallest residual error as the R-factor. The best LCF model was selected when R-factor could not be reduced by more than 20% of the previous best model. Quantification error from LCF is commonly reported and estimated to be ±10% [17]. Identification between adsorption species [18] and amorphous phases [19] require EXAFS measured to 16 Å^−1^ at a high signal to noise ratio, which was not feasible in our study. Therefore, species abundance of individual As adsorption standards and amorphous ferric arsenates were summed into general groups that cannot be resolved with our data [10]. These groups are As(III) adsorbed, As(V) adsorbed, and ferric arsenates the species included in each group are indicated in Table 1. All As XAS spectra (XANES and EXAFS) of standards and soils are in the supplemental information in Figures S1 and S2, respectively. Reference standards used for EXAFS LCF are listed in Table 1, which include lab synthesized and natural minerals received from the Smithsonian National Museum of Natural History and Excalibur Minerals Inc. (Charlottesville, VA). Natural minerals were verified using Energy Dispersive Spectroscopy or X-ray diffraction.

### 2.3 Statistical Analyses

Uncertainty for both the mouse and swine RBA As values were calculated using Fieller’s Theorem to produce 90% confidence intervals (CI) [20]. All other statistical analyses were performed using Minitab 17.2.1 and Microsoft Excel [21].

## 3. Results

### 3.1 Arsenic Speciation

The results from EXAFS LCF models of the 27 study soils and solid wastes are presented in Table 2. The major species across all soils and solid wastes was the As(V) adsorbed species group which included As(V) sorption to Al/Fe/Mn oxides. These phases are major components of As in most oxidized environmental media. Arsenic adsorbed to metal-oxide surfaces have been commonly identified via As XAS in both soil (Manning 2005; Deschamps et al. 2003; Reynolds et al. 1999; and Luo et al. 2006) and solid waste (Arcon et al. 2005; Beulieu and Savage 2005; Ritchie et al. 2013). In addition, As(V) was identified in all of the study materials ranging from 10–100%, although some (6) soils and solid wastes did contain As(III) ranging between 7 – 90%. All but two of the study materials contained the majority of As in the +5 oxidation state. Material 1 contained a very large amount (90%) of adsorbed As(III) and material 36 also contained a large amount (78%) of arsenopyrite (As (-I)).

As expected, we observed trends in As speciation that followed each sample’s contamination source. Soils that were spiked with aqueous arsenic showed As associated with highly-available, amorphous ferric arsenates and As(V) adsorbed species. The arsenic in material 16 was identified as 30% arseniosiderite, which is a calcium iron arsenate. The identification of arseniosiderite is supported by the material’s high pH (greater than 7.0) and relatively high total content of Ca and Fe (Table S4).

In pesticide-contaminated samples (1, 2, 3, 7, 18, 19, 20, and 21) the majority of the As was As(V) adsorbed species. Almost all of the arsenic existed as As(V) adsorbed species, with the exception of material 1 with an unusually high amount of As(III), fit as 90% As(III) adsorbed species. Material 7 contained 100% As(V), however it was not identified as As(V) adsorbed species, but as arseniosiderite (54%) and As(V) coprecipitated with calcite (32%).

The mining-contaminated samples (6, 8 – 13, 17, 33 – 38) had the greatest amount of reduced As, a greater variety in As oxidation states as well as a greater proportion of As-containing minerals of the samples in the dataset. As(III) was present in 5 of 14 materials as As(III) adsorbed to mineral surfaces. Samples 36 and 38 were the only materials where As(-I) was identified. Arsenopyrite (70%) was identified in sample 36Sample 38 contains As(-I) as arsenopyrite (19%).

The two samples (29 and 30) that were contaminated due to glass manufacturing did not have an observed trend associated with the contamination source. Sample 29 was best identified as predominantly amorphous ferric arsenate (63%) whereas sample 30 contained 36% amorphous ferric arsenate, 41% As(V) coprecipitated with calcite, and 23% adsorbed As(V)species.

## 4. Discussion

### 4.1 Arsenic Speciation and Bioavailability

In 2012, the U.S. Environmental Protection Agency (U.S. EPA) published a review of As bioavailability data that determined for most soils a bioavailability of 60% would not be exceeded [22]. This trend also proved to be true for the study soils and solid wastes presented here, except for the spiked materials which had RBA As ranging from 70 – 80%. The high bioavailability is most likely due to the As adsorbed to amorphous species and arseniosiderite present in the soil. Meunier et al. [12] reported that soils with high amounts of Ca-Fe-arsenates had high As bioaccessibility values due to the high solubility of these mineral phases.

The amorphous ferric arsenates species have particular importance in many natural systems and are important in mining-impacted environments where relatively high dissolved concentrations of arsenate and other cations and anions can form metal arsenate precipitates. Amorphous ferric arsenates can have significant substitution with anions (PO_4_, SO_4_, CO_3_) and cations (Ca, K, Fe, Mg). As shown within this data set, samples 9, 10, 12–17, 29, 30, 34, 37, and 38 all contain ferric arsenates but have RBA As ranging from 6 to 48% and 10 to 81% as determined using the swine and mouse bioassays, respectively. Samples 9, 10, 13, 30, 34, 37 and 38 have RBA As that are less than 26%, but samples 12, 14–17, and 29 all have RBA As that are greater than 40%. Samples with ferric arsenates and lower RBA As (9, 10, 12–17, 29, 30, 24, 27) are most likely to be a more insoluble forms of ferric arsenate. Samples with ferric arsenate and higher RBA As (12, 14–17, 29) are more likely to be soluble phases. In addition, the mineral solubilities of scorodite and yukonite were compared and show that the bioaccessible As for yukonite is much higher than that for scorodite [23].

The RBA As for the pesticide contaminated materials ranged from 20 – 46% using the adult mice bioassay and from 31 – 54% for the juvenile swine bioassay. These are midrange RBA values, which are also consistent with what Ruby et al. [3] reported. The RBA As for sample 36 was 4.0% as determined using the juvenile swine bioassay. The low RBA As associated with materials that contain high amounts of arsenopyrite is consistent with Ruby et al. [3] and Meunier et al. [12] mainly due to the low solubility of arsenopyrite. The RBA As for sample 38 was 23% as determined using the juvenile swine bioassay. Compared to material 36 the smaller amount of As(-I) and presence of more soluble sorbed As species is the cause for higher RBA As in sample 38 compared to sample 36.

Sample 29 was obtained on the site of the glass manufacturing facility whereas sample 30 was obtained from a residential area nearby. The high pH and relatively high RBA As (48%) for sample 29 is consistent with ferric arsenates. The decreased RBA As for sample 30 (26%) compared to sample 29 (48%) is likely attributed to the sorption of As on iron oxide surfaces which has lower solubility compared to ferric arsenates [3,12]. Sample 30 is a residential soil that contains more Fe oxides than sample 29.

Meunier et al. [12] compared their bioaccessibility data along with the As speciation and solubility of those mineral phases to determine a qualitative ranking (Table 3). The trend observed for the mice and swine RBA reported in this manuscript is not a clear as that presented in Meunier et al. [12]. In general, soils and solid wastes that contained arsenopyrite had the lowest bioavailability compared to the rest of the soils and solid wastes.

### 4.2 Species Groupings via Principle Component Analysis

**Fig. 1.**
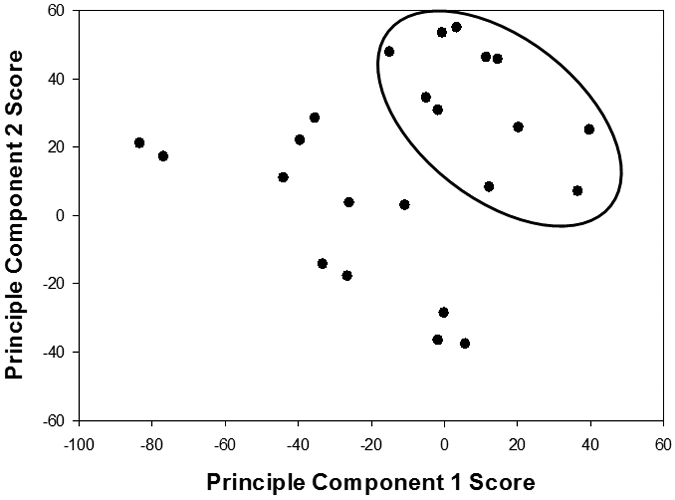
Groupings Determined via Principal Component Analysis for all 27 Soils and Solid Wastes. Solid line ferric arsenate (scorodite, kankite, and amorphous ferric arsenate). Small dotted line As(V) adsorbed species. .

The As species groups and data obtained from linear combination fitting of As speciation results were used to determine which soils and solid wastes were most similar to one another using principal component analysis (PCA). For the PCA analysis the ferric arsenate group and amorphous ferric arsenate group were summed for each material and viewed as one As speciation grouping. Figure 1 shows the score plot of principal component two versus principal component one for the As speciation data set. These two principal components described about 667% of the variance within the As data. Ferric arsenates and As(V) adsorbed species were identified as dominant As species by PCA with principal component one coefficients of 0.481 and −0.833, respectively. Principal component two included ferric arsenates and As(V) coprecipitated with jarosite with coefficients of 0.725 and −0.558, respectively. There were two major groupings within the data set which comprised of soils and solid wastes that As speciation was dominated by As(V) adsorbed species (3, 16, 18, 19, 20, 21, 33 and 38), soils and solid wastes that As speciation was dominated by ferric arsenates (9, 10, 12, 13, 14, 15, 17, 29, 30, 34, 37).

Trends were observed when comparing As speciation across As contamination source. However, comparing As contamination source within the PCA groupings does not show a trend. Soils and solid wastes that were contaminated with As due to general pesticide use, pesticide use in orchards, mining activities, tailings, and glass works were found in a mixture in both groups when only As speciation is considered. This further suggests that material type governs As speciation within soils and solid wastes. This was also shown by Meunier [24] using PCA on total elemental analysis and bioaccessibility tests and found that the soils grouped together based on location and not concentration. Materials found within the same location are expected to have similar physical and chemical properties i.e. similar type of material. Within the groupings, there was a wide range of bioaccessibility values, which was also seen in this study when comparing soil groupings, and examining the range of As RBA within PCA groups [24].

The first principal component (As(V) adsorbed and ferric arsenates) explained 47% of the variance within the As mineralogy, which follows the findings of others that As in soil closely associates with major soil oxide minerals [25]. Adding principal component two increased the variance accounted for to 67%, suggesting principal component two (As(V) coprecipitated with jarosite) is also an important species when looking at As mineralogy in soils. Although the majority of the variance is accounted for when considering ferric arsenates and As(V) adsorbed species, the inclusion of As(III) phases increases the variance accounted for to 80%, suggesting that As(III) plays a minor role in As speciation in oxidized environments.

### 4.3 Predicting Bioavailability using Arsenic Speciation

Multiple linear regression (MLR) was to generate a relationship between As mineral species (groupings) and bioavailability. Data for this study was obtained using both the adult mouse and the juvenile swine bioassays and each animal was considered separately. The regression equations for each animal are shown below (Equations 2 and 3).

(2) Mouse RBA=36.165+(0.459×Arseniosiderite) -(0.255×Ferric Arsenates)+(0.290×Am. Ferric Arsenate) -(0.201×As(V) coppt. w/ Jarosite) -(0.369×As(V) coppt. w/Calcite) -(0.160×As(III) adsorbed)+(0.00×As(V) adsorbed,

(3) Swine RBA=38.345−(0.491×Arsenopyrite)+(0.0408×Arseniosiderite) -(0.247×FerricArsenates) -(0.287×Am.Ferric Arsenate)+(0.0233×As(V) coppt. w/Jarosite)+(0.0708×As(V) coppt. w/ Calcite)+(0.321×As(III) adsorbed)+(0.00×As(V) adsorbed),

The predicted RBA As using the MLR equation for each animal bioassay compared against the actual measured RBA is shown in Table 4. The MLR for the adult mouse model was not significant with F>0.05 and the MLR for the juvenile swine model was not significant F>0.05. The MLR had an adjusted R^2^ of <0.000 and 0.101 for the adult mouse and juvenile swine bioassays, respectively.

The ability of MLR to predict RBA As was assessed by the predicted RBA being within the 90% confidence interval (CI) for the measured RBA value. However, for regulatory purposes a conservative estimate is preferred to ensure a conservative risk assessment for a contaminated area, which is defined as a predicted RBA value that is greater than or equal to (≥) the measured RBA value.

Three of the 19 predicted RBA As values (16%) generated using the MLR fell within the 90% CI of the actual RBA for the adult mouse model. The source type of As contamination does not impact the predictability of the MLR for a specific material. Arsenic speciation is not highly predictive of RBA As using the MLR. Ten of the 19 predicted RBA As (53%) using the adult mouse MLR were conservative estimates of RBA As. The MLR was not significant, with the adjusted R^2^ of <0.001 and an R^2^ value of 0.29. Bradham et al. [7] reported a significant correlation (P<0.10) between mice RBA As and arsenopyrite, however the R^2^ value that was reported was 0.28. Other correlations with As speciation were reported in Bradham et al. [7] however RBA As data was only significantly correlated with arsenopyrite. Although, the values reported between this study and Bradham et al. [7] were similar the overall variance left unexplained in both studies is too high and suggests that As speciation alone cannot be used to predicted bioavailability.

Six of the 22 predicted RBA (27%) values generated using the MLR fell within the 90% CI of the actual RBA As for the juvenile swine model. A conservative estimate of RBA As was predicted for 12 of the 22 predicted RBA (55%) values using the juvenile swine model. The majority of the soils and solid wastes that were contaminated due to mining and smelting activities had predicted RBA As values that were greater than or fell within the 90% confidence interval of the measured values. Arsenic speciation is not highly predictive of swine RBA As using MLR. Although the MLR was not significant (adjusted R^2^ 0.101 and the R^2^ (0.40) only explained approximately 40% of the total variance, the predicted RBA provided a conservative estimate of RBA As for 12 of the 22 soils. The high unexplained variance within the MLR suggests that As speciation alone cannot be used to predict bioavailability. Brattin et al. [9]used maximum likelihood estimation (MLE) to determine if adding As speciation into a predictive equation with *in vitro* bioaccessibility would improve the prediction of RBA As using the juvenile swine model for 20 soils and results showed that the R^2^ increased. To investigate the reproducibility of the As speciation data Brattin et al. [9] conducted a round robin study with 3 different laboratories and determined that the results obtained using As speciation from electron microprobe analysis were too variable and should not be included in a predictive equation for RBA As.

## 5. Conclusions

The relationship between As speciation and bioavailability in contaminated soils is complex and intertwined. The soils contained a variety of contamination sources, As speciation, and a wide range of RBA arsenic. Bioavailability was closely tied to Fe chemistry in the soils evidenced by the most abundant As species being As(V) adsorbed species. Other identified As species that closely related to As chemistry and bioavailability were ferric arsenates and As(III) minerals. The RBA As for species followed the trend that arsenopyrite was the least bioavailable compared to other As species. Despite As(V) adsorbed to common soil mineral surfaces being a major component of most soils, these soils had RBA As that ranged from 4 – 80%. This large range indicates that there is significantly different chemical behavior between sorption species in this group and this may show the importance of coupling soil properties and mineralogy to predict As bioavailability. Trends were observed in As speciation by contamination source, however the soil type, or properties is the dominant factor controlling As speciation and in turn, RBA in soils. Multiple linear regression using As speciation can provide conservative estimates of As RBA for select soils; however, it alone is not predictive of RBA As overall. Arsenic speciation is an important component of predicting As mobility and toxicity but should be considered with other soil properties to be predictive of risk and As bioavailability from soils.

## Supplementary Material

Sup Figure S1: List of As EXAFS spectra used in PCA and LCF
Table S1: As species used for XANES LCF fitting
Table S2: PCA SPOIL values and parameters from EXAFS LCF fitting
Table S3: Arsenic Oxidation States from XANES LCF results
Table S4: Physical and Chemical Properties of Study Materials.

## Figures and Tables

**Table 1.** List of Natural and Synthetic As species used for Linear Combination Fitting (LCF) to Predict As Phases in the Soil and Solid Waste Samples

As species	Molecular Formula	As Oxidation and Covalence Type
Arsenopyrite	FeAsS	As(−1)
Arsenite coppt with pyrite (syn)	FeS_2_-As	
Loellingite	FeAs_2_	
Orpiment	As_2_S_3_	As(III)-S
Realgar	As_4_S_4_	
Arsenolite	As_2_O_3_	As(III)-O
As (III) ads^1^ Ferrihydrite (syn^2^)^3^	FeOOH•0.4(H_2_O)-As(III)	
As(III) ads Al_2_O_3_ (syn)^3^	Al_2_O_3_-As(III)	
As(III) ads Montmorillonite (syn)^3^	(Na,Ca)_0.33_(Al,Mg)_2_ (Si_4_O_10_)(OH)_2_· nH_2_O- As(III)	
Arseniosiderite	Ca_2_Fe_3_(AsO_4_)_3_O_2_•3H_2_O	As(V)-O
Pharmacosiderite	KFe_4_(AsO_4_)_3_(OH)_4_ •6H_2_O	
Scorodite^4^	FeAsO_4_ •2H_2_O	
Parascorodite^4^	FeAsO_4_ •2H_2_O	
kankite^4^	FeAsO_4_ •3.5H_2_O	
Amorphous ferric arsenate (syn)	FeAsO_4_ •4–7H_2_O	
Arsenate coppt with jarosite (syn)	Na,KFe_3_(SO_4_)_2_(OH)_6_-As(V)	
Arsenate coppt with calcite (syn)	CaCO_3_-As(V)	
Lead Arsenate	PbHAsO_4_	
As (V) ads Goethite (syn)	*α*- FeO(OH)-As(V)	
As (V) ads Ferrihydrite (syn)	FeOOH•0.4(H_2_O) As(V)	
As (V) ads Birnessite (syn)	MnO_2_-As(V)	
As(V) ads Gibbsite (syn)	Al(OH)_3_-As(V)	

1 ads adsorbed

2 syn synthetic

3 As(III) sorption group

4 Ferric arsenates group

5 As(V) sorption group

**Table 2:** Arsenic speciation results

Sample	As Source	total As (mg kg^−1^)	Mouse RBA	Swine RBA	Arseno pyrite	arsenio siderite	Ferric arsenate (scorodite, kankite)	Am. ferric arsenate	As(V) coppt jarosite	As(V) coppt calcite	As(III) adsorbed	As(V) adsorbed	R-fac tor
33	Au mining	302	8.55	23.7							7	93	0.060
37	Au mining	370	9.83	11.7				54	30			16	0.084
35	Au mining	633	16.1	19.2					46			54	0.046
34	Au mining	2541	6.37	15.3				38	42			20	0.029
36	Au mining	10482		4	70							30	0.158
38	Au mining	12041		23	19		24					56	0.026
30	Glass Works	3996		26				36		41		23	0.167
29	Glass Works	4553		48				63				37	0.057
11	Mining	249	44.8	60					75			25	0.026
6	Mining	839		41.7					42		12	46	0.032
12	Mining	1236		39.7			63				13	24	0.137
10	Mining	3913	12.9	19			64				15	21	0.012
13	Mining	12483		7.87				63	37			0	0.147
3	Pesticide	222	43.5									100	0.110
18	Pesticide	283	30	31								100	0.114
7	Pesticide	332	34	54.3		54				32		14	0.020
19	Pesticide	353	46.1	41								100	0.105
21	Pesticide	375	39.4	53								100	0.329
20	Pesticide	391	21.5	49								100	0.259
1	Pesticide	464	20.2								90	10	0.418
2	Pesticide	641	29.1	39.5						47		53	0.037
8	Smelter	162	29.9	54.9					47		41	12	0.063
16	Spiked	226	81.2			30		14				56	0.093
14	Spiked	238	79.7					52				48	0.072
15	Spiked	259	69.7					66				34	0.149
17	SRM	1540	41.4	41.8			23	26	51			0	0.040
9	Tailings	521		14		32	39					29	0.062

**Table 3.** Arsenic Mineralogy Speciation Phases and Bioaccessibility Trend

	Species Group	Mineral Phase	
1	Sulfides	Arsenopyrite Realgar Pyrite	Least Bioaccessible
2	Iron Arsenates	Scorodite Kankite Pharmacosiderite Amorphous	
3	Arsenic bearing Iron(oxy) Hydroxides	Goethite Lepidocrocite Akaganeite Amorphous	
4	Roaster Iron Oxides	Hematite Maghemite	
5	Sulfates	Tooeleite Jarosite Schwertmannite	
6	Clay minerals – Generally Iron Bearing	Undifferentiated	
7	Calcium-Iron Arsenates	Yukonite amorphous	Most Bioaccessible

Adapted from Meunier et al. [12]

**Table 4.** Predicted RBA and Comparison to Actual RBA As for MLR using Arsenic Speciation Data

	Mouse RBA (%)			Swine RBA (%)		
ID	Mean	CI^a^	Predicted RBA	Mean	CI^a^	Predicted RBA
1	20.2^+^	18.1, 22.4^+^	21.8^*#^			
2	29.1^+^	26.0, 32.3^+^	18.8	39.5^+^	35.8, 43.1^+^	41.7^*#^
3	43.5^+^	37.9, 49.2^+^	36.2			
6				41.7	34.5, 48.8	43.2^*#^
7	34.0^+^	29.8, 38.3^+^	49.1^#^	52.3^+^	54.3, 58.4^+^	42.8
8	29.9^+^	26.6, 33.3^+^	20.2	54.9^+^	50.4, 59.4^+^	52.6^*^
9				14	13, 15	30.0^#^
10	12.5^+^	2.57, 22.4^+^	17.4^*#^	19	17, 20	27.4^#^
11	44.8^+^	41.6, 48.2^+^	21.1	60	56, 65	40.1
12				39.7	38.7, 40.7	27.0
13				7.87	4.33, 11.4	21.1^#^
14	79.7^+^	73.8, 85.9^+^	51.2			
15	69.7^+^	65.9, 73.6^+^	55.3			
16	81.2^+^	70.9, 91.7^+^	54.0			
17	41.4^+^	39.1, 43.6^+^	27.6	41.8	39, 45	26.4
18	30.0^+^	27.4, 32.7^+^	36.2^#^	31	25, 38	38.3^#^
19	46.1^+^	41.8, 50.5^+^	36.2	41	38, 44	38.3^*^
20	21.5^+^	17.6, 25.3^+^	36.2^#^	49	42, 57	38.3
21	39.4^+^	36.1, 42.8^+^	36.2^*^	53	49, 57	38.3
29				48	45, 51	20.3
30				26	24, 28	30.9^#^
33	8.55^+^	6.51, 10.6^+^	35.0^#^	23.7	10.9, 36.5	40.6^#^
34	6.37^+^	5.33, 7.43^+^	38.7^#^	15.3	11.7, 18.8	28.4^#^
35	16.1^+^	15.2, 17.0^+^	26.9^#^	19.2	16.9, 21.4	39.4^#^
36				4	3.3, 4.6	4.00^*#^
37	9.83^+^	8.82, 10.9^+^	45.8^#^	11.7	8.3, 15.2	23.5^#^
38				23	17.6, 28.5	23.1^*#^

a CI 90% Confidence Interval except for soil 17 (95%)

* Soils that the predicted RBA falls within the CI of the measured RBA

# Predicted RBA values that are ≥ the measured RBA
