# Supplementary material for: Arsenic Speciation of Contaminated Soils / Solid Wastes and Relative Oral Bioavailability in Swine and Mice
Source: Soil Syst. Author manuscript; Available in PMC 2019 Jul 2. (PMC6605063; doi:10.3390/soilsystems2020027)
Supplement: Sup — Figure S1: List of As EXAFS spectra used in PCA and LCF Table S1: As species used for XANES LCF fitting Table S2: PCA SPOIL values and parameters from EXAFS LCF fitting Table S3: Arsenic Oxidation States from XANES LCF results Table S4: Physical and Chemical Properties of Study Materials. [file NIHMS1525147-supplement-Sup.pdf]

Supplemental information

Linear combination fitting (LCF) for oxidation state were analyzed in the XANES over the energy range -20 to 50 eV relative to e0 with energy resolution of 0.5 eV. Quantification error from LCF is estimated to be ±10%, although actual fit error can depend on sample composition [16]. Lower error (<5%) has been reported for mixtures of As(III) and As(V) due to the significant edge energy separation (>3 eV, with energy resolution of 0.5 eV). This trend was observed in the standards used for our dataset (Table S1).

Table S1. List of As species used for XANES Linear Combination Fitting (LCF) for relative abundance of oxidation state and covalence type in the Soil and Solid Waste Samples

As species	Molecular Formula	E_0 energy (eV)	As Oxidation and Covalence Group
Arsenopyrite	FeAsS	11867.5	As(-1)
Loellingite	$FeAs_2$	11867.6	
Orpiment	As_2S_3	11868.1	As(III)-S
Realgar	As_4S_4	11868.7	
Arsenolite	As_2O_3	11871.5	As(III)-O
As (III) ads Ferrihydrite (syn[2])	$FeOOH \bullet 0.4(H_2O)$-As(III)	11871.0	
As(III) ads Al_2O_3 (syn)	Al_2O_3-As(III)	11871.0	
As(III) ads Montmorillonite (syn)	$(Na,Ca)_{0.33}(Al,Mg)_2(Si_4O_{10})(OH)_2 \cdot nH_2O$- As(III)	11870.9	
Arseniosiderite	$Ca_2Fe_3(AsO_4)_3O_2 \bullet 3H_2O$	11874.3	As(V)-O
Pharmacosiderite	$KFe_4(AsO_4)_3(OH)_4 \bullet 6H_2O$	11874.3	
Scorodite	$FeAsO_4 \bullet 2H_2O$	11874.3	
Amorphous ferric arsenate (syn)	Am-$Fe_{12}(AsO_4)_{10}(OH)_{20} \bullet 15H_2O$	11874.4	
Lead Arsenate	$PbHAsO_4$	11873.8	
As (V) ads Goethite (syn)	α- FeO(OH)-As(V)	11874.2	
As (V) ads Ferrihydrite (syn) (low As)	$FeOOH \bullet 0.4(H_2O)$ As(V)	11874.4	
As (V) ads Ferrihydrite (syn)	$FeOOH \bullet 0.4(H_2O)$ As(V)	11874.2	
As (V) ads Hematite (syn)	Fe_2O_3-As(V)	11874.3	
As (V) ads Birnessite (syn)	MnO_2-As(V)	11874.4	
As(V) ads Gibbsite (syn)	$Al(OH)_3$-As(V)	11874.4	

[1]ads adsorbed [2]syn synthetic

Table S2 Principal component analysis SPOIL values and parameters for EXAFS LCF

As species	Group 1	Group 2	Group 3	Group 4	Group 5
Arsenopyrite	46.53	1.09	54.05	26.79	95.33
Arsenite coppt with pyrite (syn)	25.48	2.78	14.07	3.43	53.48
Loellingite	34.77	4.54	19.74	5.17	70.10
Orpiment	37.80	2.53	14.19	3.24	80.28
Realgar	152.58	2.42	16.86	5.05	234.28
Arsenolite	28.36	12.80	1.04	1.84	64.41
As (III) ads[1] Ferrihydrite (syn[2])	11.01	23.96	3.50	1.31	20.57
As(III) ads Al_2O_3 (syn)	5.22	13.19	3.50	0.94	9.56
As(III) ads Montmorillonite (syn)	7.45	16.59	3.25	1.12	14.13
Arseniosiderite	0.77	2.78	1.57	2.40	2.13
Pharmacosiderite	1.60	3.80	2.93	3.27	3.55
Scorodite	1.30	4.33	2.00	2.65	2.44
Parascorodite	0.93	3.23	1.39	3.24	2.19
kankite	0.67	2.55	1.04	1.48	1.70
Amorphous ferric arsenate (syn)	1.14	3.01	2.12	3.10	1.80
Arsenate coppt with jarosite (syn)	0.82	2.16	1.95	2.39	2.23
Arsenate coppt with calcite (syn)	0.68	2.75	1.33	3.76	1.57
Lead Arsenate	0.92	2.94	1.71	3.60	1.66
As (V) ads Goethite (syn)	2.60	3.40	4.39	3.58	4.97
As (V) ads Ferrihydrite (syn)	1.33	2.52	2.16	2.63	2.65
As (V) ads Birnessite (syn)	1.37	4.27	3.21	4.84	2.30
As(V) ads Gibbsite (syn)	1.17	2.52	2.05	2.54	1.88

[1]ads adsorbed [2]syn synthetic

Soils in groups were selected based on similar contamination source. Group 1 contains soils contaminated with lead arsenate pesticide, samples 18, 19, 20, 21. Group 1 was fit over 3-11 $Å^{-1}$ with k-weight of 3 and two significant components. Group 2 contains soils from gold mining contamination; samples 33, 34, 35, 36, 37, 38. Group 3 contains misc. orchard contamination, samples 1, 2, 3, 7. Group 4 contains misc. mining, samples 6, 8, 9, 10, 11, 12, 13, 17. Group 5 contains glass works and NaAs salt spikes, samples 14, 15, 16, 29, 30.

Table S3. Arsenic Oxidation from XANES linear combination fitting Results

ID	As(-I)	As(III)-S	As(III)-O	As(V)-O	R-factor
1	0	0	76	24	0.000200
2	0	0	0	100	0.000723
3	0	0	0	100	0.000197
6	0	03	90	88	0.000192
7	0	0	0	100	0.000371
8	0	0	47	53	0.000347
9	0	0	0	100	0.000327
10	0	11	0	89	0.002712
11	0	10	0	90	0.000269
12	0	0	9	78	0.001586
13	0	0	0	100	0.005882
14	0	0	0	100	0.002945
15	0	0	0	100	0.000122
16	0	0	0	100	0.000215
17	0	0	8	91	0.006647
18	0	0	0	100	0.000086
19	0	0	0	100	0.000151
20	0	0	0	100	0.000207
21	0	0	0	100	0.000136
29	0	0	0	100	0.003222
30	0	0	0	100	0.003529
33	0	10	0	90	0.000115
34	0	0	0	37	0.000963
35	0	0	0	100	0.001955
36	78	0		22	0.000435
37		3		97	0.000286
38	22	0	0	35	0.000965

All values are reported in % [1] ads adsorbed

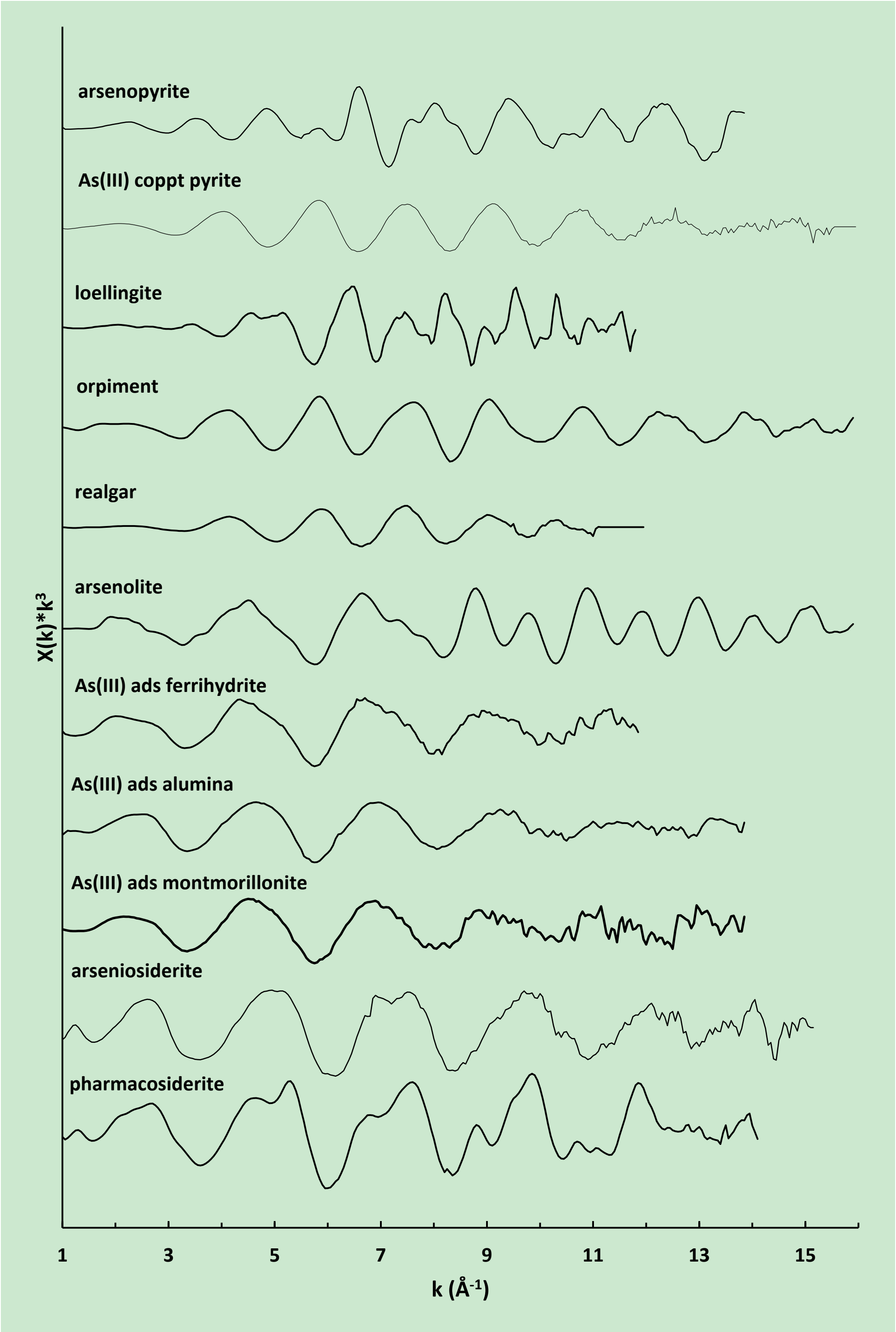
arsenopyrite
As(III) coppt pyrite
loellingite
orpiment
realgar
arsenolite
As(III) ads ferrihydrite
As(III) ads alumina
As(III) ads montmorillonite
arseniosiderite
pharmacosiderite
X(k)*k3
1
3
5
7
9
11
13
15
k (Å-1)

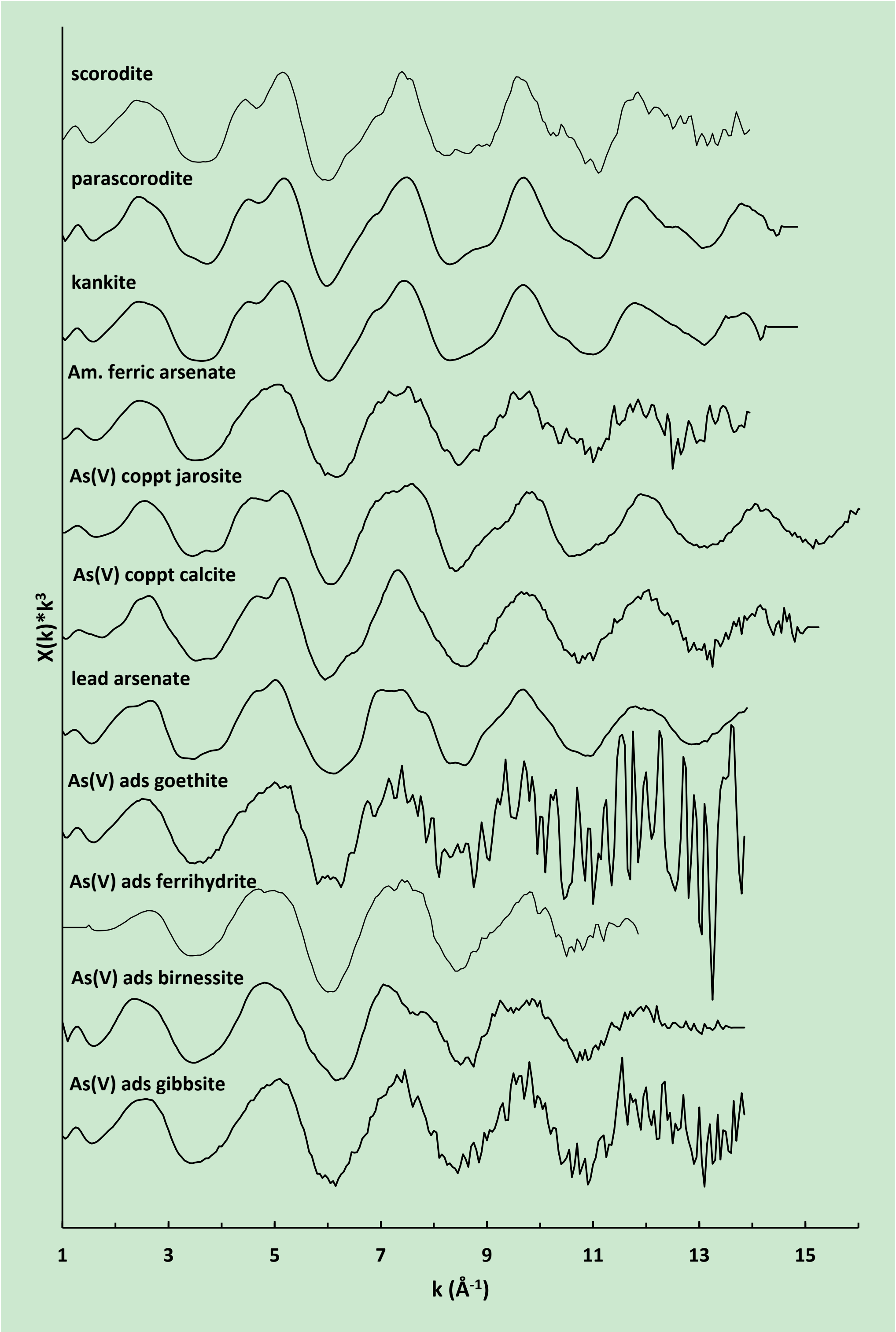

Figure S1 List of As K-edge normalized, k^3 weighted EXAFS spectra used in PCA and LCF of soils and solid wastes

Table S4 Physical and Chemical Properties of Study Soils and Solid Wastes

ID	As (mg/kg)	Al (g/kg)	Fe (g/kg)	CaO (%)	pH
1	464	60	118	3.98	7.34
2	641	62	82	3.40	5.61
3	222	39	40	1.90	6.67
6	839	8	31	0.600	6.84
7	332	50	35	1.10	6.07
8	162	29	28	3.00	7.30
9	521	14	17	7.30	9.28
10	3910	16	148	4.10	2.82
11	249	6	87	2.60	2.14
12	1240	14	161	11.7	7.71
13	12500	5	283	0.400	3.02
14	238	19	12	18.3	7.75
15	259	20	17	8.30	7.55
16	226	29	23	1.20	7.55
17	1540	19	43	1.30	4.00
18	283	47	29	0.400	6.01
19	353	78	48	2.30	5.80
20	391	58	42	1.60	5.42
21	375	55	40	2.30	6.22
29	4550	18	22	12.7	7.47
30	4000	17	23	4.70	8.24
33	302	72	59	1.20	7.81
34	2540	52	82	0.600	4.96
35	633	51	60	0.600	6.53
36	10500	17	56	3.30	7.63
37	370	71	57	0.200	5.78
38	12000	45	74	0.900	7.29
Minimum	162	5	12	0.200	2.14
Maximum	12500	78	283	18.3	9.28
Mean	2210	36.0	63.7	3.70	6.33
Median	464	29	43	2.30	6.67
